# Bitter Gentian Teas: Nutritional and Phytochemical Profiles, Polysaccharide Characterisation and Bioactivity

**DOI:** 10.3390/molecules201119674

**Published:** 2015-11-05

**Authors:** Daniil N. Olennikov, Nina I. Kashchenko, Nadezhda K. Chirikova, Lena P. Koryakina, Leonid N. Vladimirov

**Affiliations:** 1 Institute of General and Experimental Biology, Siberian Division, Russian Academy of Science, Sakh’yanovoy Str., 6, Ulan-Ude 670047, Russia; ninkk@mail.ru; 2 Department of Biochemistry and Biotechnology, North-Eastern Federal University, 58 Belinsky Str., Yakutsk 677027, Russia; hofnung@mail.ru; 3 Faculty of the Veterinarian Medicine, Yakut State Agricultural Academy, 15 Krasil’nikova Str., Yakutsk 677007, Russia; nir06@mail.ru (L.P.K.); vladimirov.l15@yandex.ru (L.N.V.)

**Keywords:** *Gentiana algida*, *Gentiana decumbens*, *Gentiana macrophylla*, *Gentiana triflora*, iridoids, flavonoids, polysaccharides, anti-inflammatory, antioxidant, antimicrobial activity

## Abstract

As a result of the wide distribution of herbal teas the data on nutritional characterisation, chemical profile and biological activity of these products are required. The decoctions of *Gentiana algida*, *G. decumbens*, *G. macrophylla* and *G. triflora* herb teas were nutritionally characterized with respect to their macronutrients, demonstrating the predominance of polysaccharides and low lipid content. Gentian decoctions were also submitted to a microcolumn RP-HPLC-UV analysis of phytochemicals demonstrating a high content of iridoids (177.18–641.04 μg/mL) and flavonoids (89.15–405.71 μg/mL). Additionally, mangiferin was detected in samples of *G. triflora* tea (19.89 μg/mL). Five free sugars (fructose, glucose, sucrose, gentiobiose, gentianose) were identified in all gentian teas studied, as well as six organic acids (malic, citric, tartaric, oxalic, succinic, quinic). Pectic polysaccharides with a high content of rhamnogalacturonans and arabinogalactans were also identified and characterized in gentian decoctions for the first time. Gentian tea decoctions and their specific compounds (gentiopicroside, loganic acid-6′-*O*-β-d-glucoside, isoorientin, isoorientin-4′-*O*-β-d-glucoside, mangiferin, water-soluble polysaccharides) showed a promising antimicrobial, anti-inflammatory and antioxidant potentials. Evidences obtained indicate the prospective use of gentian herb teas as food products and medicines.

## 1. Introduction

The tradition of tea beverage consumption is distributed worldwide. Nowadays, the tea drinking custom is considered to be associated with the region, environmental factors, ethnic group, *etc.* Herbal teas constitute a separate group of tea beverages. This refers to any beverage made from the infusion or decoction of herbs, spices, or other plant materials in hot water, and usually it does not contain caffeine. The consumption of herbal teas is particularly popular among the nomadic people of Siberia (Buryats, Yakutians, Evenkies, Soyots and others). Inhabitants from different tribes apply diverse tea beverages made with an eclectic assortment of ingredients, including local raw materials. Some herbal teas are used for curative purposes. Siberian nomads have a special category of such decoctions known as bitter teas which are made chiefly from the gentianaceous plants. Such teas are widely used because of the predominance of proteins in the diet and used as a remedy in various digestive disorders ranging from simple types such as vomiting to more complex problems like dyspepsia [1,2,3,4].

Despite the archaic manner of application of bitter decoctions, it is still the customary to consume bitter herbal teas before meals as specific aperitifs. Information on the gastric stimulatory effects of four frequently used Siberian gentian herbs (*Gentiana algida*, *G. decumbens*, *G. macrophylla*, *G. triflora*) has been presented earlier, and their effectiveness has been demonstrated [5]. In several traditional medical systems (Traditional Chinese Medicine, Traditional Tibetan Medicine) decoctions of these gentians are also used as anti-inflammatory, antimicrobial, wound-healing agents, *etc.* [1,3,6,7]. The known literature data on chemical composition and biological activities relates primarily to the roots of the gentians but little information is available about the herbs [8,9], confirming the poor general knowledge level of the nutritional, chemical, and pharmacological data of decoctions from the herbs *G. algida*, *G. decumbens*, *G. macrophylla* and *G. triflora*, and suggesting a need for further work in this direction.

In addition to the known low-molecular weight compounds, an investigation of the chemical composition and biological activity of the polymeric plant compounds is of particular interest. As water is a universal extractant that allows dissolution of most plant polymers, polysaccharides are therefore usual components of any plant-derived tea product. According to known data, in the *Gentiana* genus only *G. rigescens* [10] and *G. scabra* root polysaccharides [11] have been previously characterised. To the best of our knowledge, no information about the chemical composition and bioactivity of polysaccharides of the *Gentiana* herbs has been previously reported.

In the present work, the nutritional characterisation, and free sugars and organic acids profiling of *G. algida*, *G. decumbens*, *G. macrophylla* and *G. triflora* herbal teas were performed, and gentian decoctions were submitted to a detailed analysis of phytochemicals (iridoids, phenolic compounds, polysaccharides). Antimicrobial, anti-inflammatory, and antioxidant activities of gentian tea preparations and their representative phytochemicals were investigated.

## 2. Results and Discussion

### 2.1. Nutritional Profiles of Bitter Gentian Teas Decoctions

The results of the nutritional characterisation of the decoctions of four bitter gentian teas including organoleptic parameters (colour, odour, taste) and nutritional compounds (macronutrients, free sugars, organic acids) are presented in Table 1.

**Table 1:** Organoleptic and nutritional characteristics of four gentian tea decoctions ^a^.

Parameter	*G. algida*	*G. decumbens*	*G. macrophylla*	*G. triflora*
Extractives ^b^	272 ± 8	224 ± 8	263 ± 9	345 ± 12
*Organoleptic characteristics*				
Color	Light-brown	Yellow	Yellow	Dark-yellow
Odor	Herbal	Herbal	Herbal	Herbal
Taste	Bitter, intensive	Bitter, light	Bitter, light	Bitter, medium
Bitter index ^c^	9300 (4200–12,000)	500 (170–600)	1000 (520–1200)	3050 (1300–4800)
*Nutritional characteristics*				
Carbohydrates ^b^	98.39 ± 3.05	95.23 ± 2.76	107.11 ± 3.42	151.99 ± 4.71
Protein ^b^	22.65 ± 0.77	22.75 ± 0.68	40.01 ± 1.12	30.91 ± 0.95
Lipids ^b^	<0.1	<0.1	<0.1	<0.1
Ash ^b^	8.63 ± 0.43	9.39 ± 0.49	5.18 ± 0.23	6.47 ± 0.32
Energy ^d^	0.48	0.47	0.59	0.73
Total free amino acids ^b^	2.39 ± 0.06	1.37 ± 0.03	3.84 ± 0.11	2.19 ± 0.06
*Free sugars*				
Fructose ^b^	2.00 ± 0.05	8.18 ± 0.20	1.42 ± 0.04	7.42 ± 0.22
Glucose ^b^	16.37 ± 0.39	32.64 ± 0.75	2.03 ± 0.05	51.57 ± 1.34
Sucrose ^b^	26.35 ± 0.63	20.26 ±0.45	24.32 ± 0.58	25.28 ± 0.61
Gentiobiose ^b^	4.16 ± 0.09	6.51 ± 0.15	9.20 ± 0.22	13.24 ± 0.30
Gentianose ^b^	5.72 ± 0.15	21.80 ± 0.59	31.93 ± 0.83	37.76 ± 1.06
Total free sugars ^b^	54.60	89.39	68.90	135.27
*Organic acids*				
Malic acid ^b^	9.64 ± 0.29	15.63 ± 0.43	5.39 ± 0.15	4.87 ± 0.11
Citric acid ^b^	4.16 ± 0.11	6.11 ± 0.15	4.83 ± 0.12	9.31 ± 0.22
Tartaric acid ^b^	0.72 ± 0.02	1.24 ± 0.02	0.94 ± 0.02	0.52 ± 0.01
Oxalic acid ^b^	tr.	tr.	0.11 ± 0.00	0.09 ± 0.00
Succinic acid ^b^	tr.	tr.	tr.	0.12 ± 0.00
Quinic acid ^b^	tr.	tr.	tr.	tr.
Total organic acids ^b^	14.52	22.98	11.27	14.91

^a^ standard brewing—1 g plant material/100 mL water; ^b^ mg/100 mL decoction; ^c^ median value (min–max values); ^d^ kcal/100 mL decoction; tr.—traces amounts (<limit of quantification).

Extractives ranged from 224 mg/g (*G. decumbens*) to 345 mg/g (*G. triflora*), demonstrating the good extractability of the gentian tea compounds by boiling water. Colour and odour of the four gentian teas were typical for herbal teas, though the tastes were characterised as having bitterness of different intensity. Bitter indices varied from 500 units in *G. decumbens* to 9300 units in *G. algida*. Generally, organoleptic characteristics allow us to describe these gentian teas as specific products with atypical but pleasant tastes and odours.

Among macronutrients, carbohydrates were the most abundant compounds, reaching 95.23–151.99 mg/100 mL decoction, followed by proteins (22.65–40.01 mg/100 mL decoction). Low lipid content (<0.1 mg/100 mL decoction), ash (5.18–9.39 mg/100 mL decoction) and energy content (0.47–0.73 kcal/100 mL decoction) were detected in the gentian teas.

Five free sugars were identified in the gentian teas, including glucose, fructose and sucrose, which were usual for plants, as well as two oligosaccharides, gentiobiose [β-d-glucopyranosyl-(1→6)-d-glucopyranose] and gentianose [β-d-glucopyranosyl-(1→6)-α-d-glucopyranosyl-(1→2)-β-d-fructofuranose], specific for the *Gentianaceae* family [12]. Sucrose was a dominant free sugar in *G. algida*, glucose in *G. decumbens* and *G. triflora*, and gentianose in *G. macrophylla*. The presence of the five mentioned sugars had been shown previously in *G. macrophylla* and *G. decumbens* flowers [12]. The determination of the free sugar composition of *G. algida* and *G. triflora* and quantification of free sugar in the four species were realised in this work for the first time.

Organic acids in the gentian teas included six compounds—malic, citric, tartaric, oxalic, succinic and quinic acids. The total content of organic acids in the teas was 11.27–22.98 mg/100 mL decoction. Malic acid was the prevalent compound in *G. algida*, *G. decumbens*, and *G. macrophylla*. In contrast, citric acid dominated in *G. triflora* tea. Malic, citric, tartaric, and succinic acids have potent biological properties like antimicrobial [13], cardioprotective [14], and antiplatelet properties [15]. The low content of oxalic acid (tr.—0.11 mg/100 mL decoction) should be noted, as it is characterised as an antinutrient compound due to its inhibitory effect on mineral bioavailability [16].

As far as we know, this is the first report on nutritional characterisation of four gentian products that proved to be equilibrated valuable herbs rich in carbohydrates and proteins, and poor in fat and calories.

### 2.2. Phytochemical Profiles of Bitter Gentian Tea Decoctions

Quantification of low-molecular weight phytochemicals in the decoctions of bitter gentian tea was realised by an microcolumn (MC)-RP-HPLC-UV procedure [17]. Previously, the presence of the 12 compounds including five iridoids (loganic acid, loganic acid-6′-*O*-β-d-glucoside, swertiamarin, sweroside, gentiopicroside), six flavone-*C*/*O*-glycosides (isoorientin, isoorientin-4′-*O*-β-d-glucoside, isovitexin, saponarin, isosaponarin, isoscoparin) and mangiferin were found in the hydro-ethanolic extracts of gentian herbs of Siberian origin [5]. The present work aimed the quantification of mentioned compounds in the gentian herb decoctions. All the contents are summarised in Table 2.

**Table 2:** Content of iridoids, flavonoids and mangiferin in bitter gentian tea decoctions, μg/mL (±SD).

Compound	*G. algida*	*G. decumbens*	*G. macrophylla*	*G. triflora*
*Iridoids*				
Loganic acid	106.42 ± 1.91	32.69 ± 0.59	40.09 ± 0.72	33.04 ± 0.66
Loganic acid-6′-*O*-β-d-glucoside	n.d.	523.10 ± 10.99	123.08 ± 2.34	592.41 ± 13.03
Swertiamarin	26.38 ± 0.47	tr.	4.02 ± 0.06	tr.
Sweroside	23.03 ± 0.46	n.d.	2.91 ± 0.05	n.d.
Gentiopicroside	259.57 ± 4.67	n.d.	7.08 ± 0.13	15.59 ± 0.34
Subtotal	415.40	555.79	177.18	641.04
*Flavonoids*				
Isoorientin	173.60 ± 2.77	14.83 ± 0.27	27.83 ± 0.53	120.22 ± 1.92
Isoorientin-4′-*O*-β-d-glucoside	tr.	40.71 ± 0.89	111.10 ± 2.22	204.43 ± 3.88
Isovitexin	37.96 ± 0.75	18.11 ± 0.34	40.04 ± 0.92	9.70 ± 0.19
Saponarin	25.62 ± 0.46	15.50 ± 0.25	6.12 ± 0.13	29.31 ± 0.59
Isosaponarin	n.d.	tr.	tr.	21.22 ± 0.45
Isoscoparin	n.d.	tr.	12.04 ± 0.26	20.83 ± 0.48
Subtotal	237.18	89.15	197.13	405.71
*Xanthones*				
Mangiferin	n.d.	n.d.	n.d.	19.89 ± 0.46
Subtotal	n.d.	n.d.	n.d.	19.89
Total	652.58	644.94	374.31	1066.64

n.d.—not detected (<limit of detection); tr.—traces amounts (<limit of quantification).

The amounts of the specific components found varied among the different gentian species. The results showed that the total content of quantifiable compounds in the gentian tea samples displayed a 2.9-fold variation. *G. triflora* tea had the highest total compound content (1066.64 μg/mL), followed in order by *G. algida* (652.58 μg/mL), *G. decumbens* (644.94 μg/mL) and *G. macrophylla* (374.31 μg/mL). Gentiopicroside was found in the highest concentration in *G. algida* tea (259.57 μg/mL), while the lowest content of the compound was detected in *G. triflora* (15.59 μg/mL) and *G. macrophylla* teas (7.08 μg/mL). Detectable quantities of sweroside and swertiamarin were found in samples of *G. algida* (23.03 and 26.38 μg/mL, respectively) and *G. macrophylla* teas (2.91 and 4.02 μg/mL, respectively). *G. decumbens* and *G. triflora* teas had the highest concentration of loganic acid-6′-*O*-β-d-glucoside, 523.10 and 592.41 μg/mL, respectively. Quantifiable prevalence of isoorientin was noted only in *G. algida* tea (173.60 μg/mL). Isoorientin-4′-*O*-β-d-glucoside was a dominant flavonoid in the other gentian teas, with the highest content in *G. triflora* (204.43 μg/mL). In the case of *G. triflora* teas, mangiferin concentrations were 19.89 μg/mL.

The data obtained allow us to calculate the total uptake of the specific compounds after application of the standard dosage of gentian teas (100 mL). It was 37.43 mg for *G. macrophylla* tea, 64.49 mg for *G. decumbens* tea, 65.26 mg for *G. algida* tea, and 106.66 mg for *G. triflora* tea. The HPLC quantification results are clear evidence that gentian tea decoctions are a good source of iridoids and phenolic compounds.

It should be noted that the iridoid content in *G. algida* and *G. decumbens* decoctions are close (415.40 and 555.79 μg/mL, respectively) but the bitter index of *G. algida* decoction is 18.6 times more that of *G. decumbens* decoction. We decided to find out the reasons for this contradiction. First we determined the bitter indices of all phytochemicals (iridoids, flavonoids and mangiferin) identified in the gentian teas. As expected, gentiopicroside was the most bitter compound, with a bitterness index of 14,500 (min–max 11,000–16,500). This parameter is slightly different from the commonly reported value (12,000) but this difference should be attributed to some subjectivity of the sensory analysis. Sweroside has a lower bitterness of 9500 (min–max 6000–12,500), followed by swertiamarin with a bitter index of 7200 (min–max 4100–8600). Structural features such as a saturated C_5_–C_6_ bond (sweroside) and the presence of a hydroxyl function at the C_5_-atom (swertiamarin) are the reasons for the decreased bitter taste. Loganic acid may be characterised as a non-bitter compound with a dominant salty taste, and loganic acid-6′-*O*-β-d-glucoside is tasteless. Neither flavonoids nor mangiferin have any taste. Therefore, only three compounds can cause influence on taste receptors—gentiopicroside, sweroside and swertiamarin.

### 2.3. Polysaccharide Characterisation of Bitter Gentian Teas

The yields of water soluble polysaccharide fractions (WSPF) were 19.34 mg/g, 6.27 mg/g, 22.18 mg/g, and 16.31 mg/g for *G*. *algida*, *G*. *decumbens*, *G*. *macrophylla* and *G*. *triflora*, respectively (Table 3).

**Table 3:** General parameters and monosaccharide compositions of water soluble polysaccharide fractions of bitter gentian teas.

Parameter	*G. algida*	*G. decumbens*	*G. macrophylla*	*G. triflora*
Yield, mg/g	19.34	6.27	22.18	15.31
Total carbohydrate content, mg/g	946.14 ± 24.59	931.63 ± 24.15	932.07 ± 22.37	954.91 ± 24.83
Uronic acid content, mg/g	802.37 ± 20.86	732.64 ± 18.31	824.63 ± 22.26	797.68 ± 20.74
Protein content, mg/g	16.27 ± 0.41	22.86 ± 0.48	21.15 ± 0.51	18.37 ± 0.38
$\alpha_{D}^{40}$, °	+116	+101	+124	+110
Reaction with I_2_	negative	negative	negative	negative
Reaction with resorcinol	negative	negative	negative	negative
Reaction with Yariv reagent	positive	positive	positive	positive
Monosaccharide composition, mol %				
Ara	9.4	8.0	4.2	10.6
Gal	8.4	12.9	7.3	7.7
Glc	2.7	7.4	1.8	1.6
Man	0.9	2.1	1.2	1.1
Rha	5.6	4.8	6.3	2.8
Xyl	1.4	0.9	0.8	1.1
GalA	71.6	63.9	78.4	75.1
GlcA	tr.	tr.	tr.	tr.

tr.—traces amounts (<limit of quantification).

All isolated WSPF were characterised by a high total carbohydrate content (931.63–954.91 mg/g) and low protein content (16.27–22.86 mg/g). High uronic acid content (732.64–824.63 mg/g) was reflected in the specific rotation values (+101–+124°) and indicated the presence of polygalacturonan compounds in the WSPF. Negative reactions with iodine and resorcinol declared the absence of starch and inulin-derived polymers. Interestingly, all WSPF gave a positive reaction with Yariv reagent, which is specific for arabinogalactan-protein complexes (AGP).

The monosaccharide compositions of the WSPF of the four bitter gentian teas demonstrated a dominance of galacturonic acid, with the smallest amount in *G. decumbens* (63.9 mol %) and largest in *G. macrophylla* (78.4 mol %) (Table 3). Content of arabinose and galactose were 4.2 mol %–10.6 mol % and 7.3 mol %–12.9 mol %, respectively. Rhamnose, glucose, xylose and mannose were detected in all samples, as well as glucuronic acid in trace levels. Generally, the monosaccharide compositions of the WSPF were typical for pectic polysaccharides containing polygalacturonans, rhamnogalacturonans and arabinogalactans [18].

**Figure 1:**
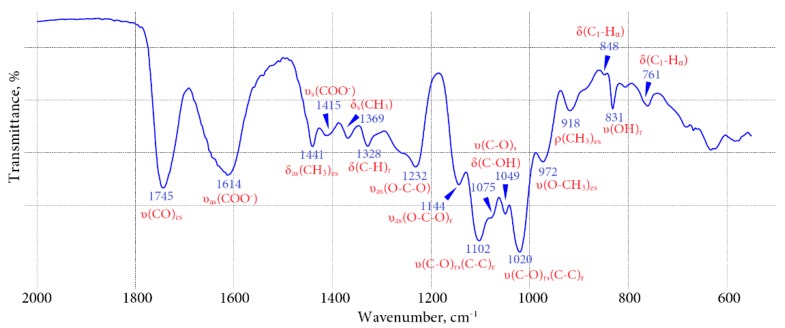
FT-IR spectra of water-soluble polysaccharide fraction of *G. algida* bitter tea.

FT-IR spectra of the WSPF of the four bitter gentian teas are very close to each other, similar to those for pectin polysaccharides [19]. The FT-IR spectrum of the WSPF of *G. algida* bitter tea is shown in Figure 1. It contains specific bands assigned to stretching C=O vibrations of esterified groups (1745 cm^−1^) and asymmetric stretching vibrations of free carboxylic groups (1624 cm^−1^) [20]. Additional bands at 1441 cm^−1^, 1415 cm^−1^, 1369 cm^−1^, and 972 cm^−1^ may be ascribed to the ester and carboxylate groups. The band at 1328 cm^−1^ was assigned to C–H bending vibrations of the pyranoid ring. The characteristic “anomeric region” bands for α-linkages at 848 cm^−1^ and 761 cm^−1^ were observed [21]. The presence of two intense bands at 1102 cm^−1^ and 1020 cm^−1^ which coincided with vibrations of glycosidic bonds and pyranoid rings was due to the high homogalacturonan content. In contrast, the low intensity of bands at 1075 cm^−1^ and 1049 cm^−1^, specific for rhamnogalacturonans, indicates their low content [22].

Based on the data obtained, we can conclude that the water soluble polysaccharides identified in the gentian bitter teas are pectins with a high content of homogalacturonan compounds as well as rhamnogalacturonans and arabinogalactans. Despite the fact that further investigations are needed to understand the structural features of gentian herb polysaccharides, as part of this work we carried out chemical examination of the four *Gentiana* herb polysaccharides for the first time. It is known that the plant pectins possess a wide spectrum of biological activity—immune-stimulating, anticancer, hypoglycaemic, anti-inflammatory and anticoagulant properties [18]. Taking into account the high therapeutic efficiency of plant pectic polysaccharides, the *Gentiana* genus might be deduced as a prospective source of biologically active polymeric nutrients.

### 2.4. Biological Activity of Bitter Gentian Teas

#### 2.4.1. Anti-Inflammatory Activity

Carrageenan-induced rat paw edema was used to study the anti-inflammatory activity of the four gentian teas. The model of inflammation caused by carrageenan is an acute and reproducible model which results in a quantifiable increase of paw size (edema) and is also modulated by inhibitors within the inflammatory cascade [23].

**Figure 2:**
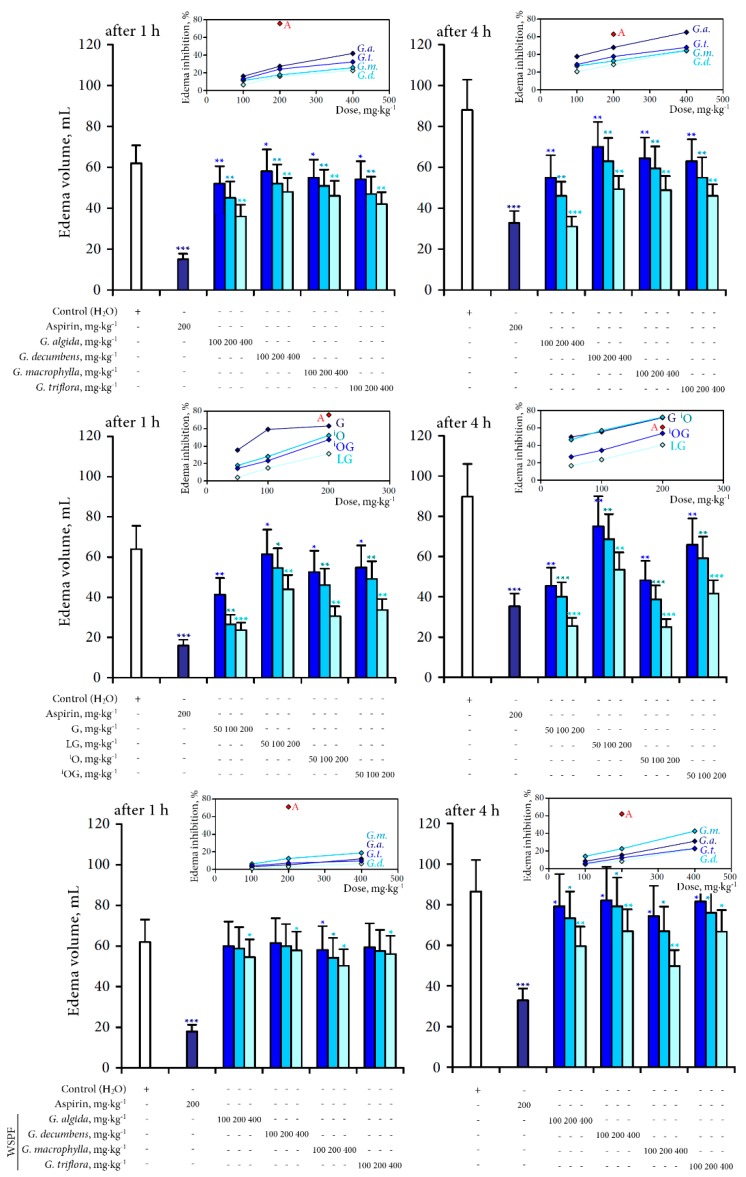
Anti-inflammatory activity of four gentian teas and individual compounds on carrageenan-induced paw edema in rats after 1 and 4 h after injection. Bars show the values of edema volume (mL). Values of edema shown are mean ± SD (*n* = 12). Asterisks indicate statistically significant values from the control (*—*p* < 0.05; **—*p* < 0.01; ***—*p* < 0.001). On cuts—percentage of edema inhibition (A—aspirin, *G.a.*—*G. algida*, *G.d—G. decumbens*, *G.m.*—*G. macrophylla*, *G.t.*—*G. triflora*, G—gentiopicroside, LG—loganic acid-6′-*O*-β-d-glucoside, ^i^O—isoorientin, ^i^OG—isoorientin-4′-*O*-β-d-glucoside, WSPF—water-soluble polysaccharide fractions).

Aspirin, a common anti-inflammatory drug, was used as a reference substance. In our results, all investigated teas administered orally in doses of 100–400 mg/kg displayed a marked and similar intensity of action (Figure 2).

All the gentian tea extracts demonstrated a dose-dependent mode of suppressive action and were able to reduce edema size after 1–4 h after injection of the inflammatory agent. The percentage of edema inhibition was more expressed for *G. algida* tea (16.1%–41.9% after 1 h, 37.5%–64.8% after 4 h) and less expressed for *G. decumbens* tea (6.5%–22.6% after 1 h, 20.3%–43.8% after 4 h). *G. algida* tea in a dose of 400 mg/kg after 4 h showed activity similar to aspirin (200 mg/kg).

Among the main phytochemicals identified in the gentian teas gentiopicroside was the most active compound reduced edema size after 1 h after injection of the inflammatory agent in doses 50–200 mg/kg at 35.47%–63.12% comparing with control. However, after 4 h activities of gentiopicroside and isoorientin were close (49.44%–71.60% and 46.33%–72.27%, respectively). The possible reason of this feature is various bioaccessibility/bioavailability values for both compounds. Gentiopicroside is a compound with high bioavailability value [24] in contrast to isoorientin characterised as low-bioavailable compound [25]. Finally, the mentioned facts may play an important role in the process of bioactivity implementation. Loganic acid-6′-*O*-β-d-glucoside and isoorientin-4′-*O*-β-d-glucoside were the compounds with a less pronounced anti-inflammatory effect.

Anti-inflammatory activity of water-soluble polysaccharide fractions of four gentians characterised as week after 1 h after injection of phlogogenic agent comparing with activity of crude decoctions and phytochemicals. Only *G. macrophylla* water-soluble polysaccharide fraction in dose 100–400 mg/kg inhibited the reduction of edema at 6.14%–18.74%. After 4 h the effectiveness of polysaccharide fractions was more expressed reached maximal values in dose 400 mg/kg—42.48% for *G. macrophylla* polysaccharides, 31.02% for *G. algida* polysaccharides, and 22.80% and 22.45% for *G. triflora* and *G. decumbens* polysaccharides, respectively.

Speculating about the possible reasons of the anti-inflammatory activity of bitter gentian teas, it can be concluded that a combination of chemical agents may be the main reason for the high activity. Particularly, the suppressive effects of gentiopicroside and swertiamarin have been demonstrated previously in an *in vitro* model system of inhibition of COX-1, COX-2 and TNF-α [26]. Loganic acid has been found to be active in a carrageenan-induced inflammation test [27]. Besides iridoids, flavonoids are known inhibitors of inflammation. The basic phenolic compound of the investigated gentian teas, an isoorientin, was previously denoted as a strong anti-inflammatory agent in carrageenan-induced inflammation in mice [28]. Also, a remarkable inhibitory effect of isoorientin on the synthesis of thromboxane B_2_ and leukotriene B_4_ has been demonstrated [29]. Isoorientin has been identified as the main active anti-inflammatory compound of *Phyllostachys edulis* (Carr.) J. Houz leaves due to its inhibitory activity on tumour necrosis factor (TNF), α-induced release of interleukins 6 and 8 and vascular endothelial growth factor [30]. Pectic substances may possess anti-inflammatory effectiveness. It is known that celery pectin in a model of lipopolysaccharide (LPS) induced inflammation can decrease interleukin-1β, increase interleukin-10 production, and diminish the amount of neutrophils migrating to the peritoneal cavity after LPS injection [31]. Pectic polysaccharide from alfalfa has been shown to have a significant anti-inflammatory effect against mRNA expression of the pro-inflammatory cytokine genes [32]. Comaruman, a pectin from *Comarum palustre* L., inhibits spontaneous and phorbol-12-myristate-13-acetate-activated adhesion of peritoneal leukocytes *in vitro* [33]. Thus, it is likely that the effectiveness of bitter gentian teas is due to their high content of phytochemicals known to have anti-inflammatory properties.

#### 2.4.2. Antimicrobial Activity

The results of the determination of antimicrobial activity of the gentian tea decoctions demonstrated that all extracts inhibited the growth of six microorganisms tested including Gram-positive/negative cultures and one fungal species (Table 4). The minimum inhibitory concentration (MIC) of *G. algida* tea was the highest with values of 100–200 μg/mL. The most sensitive to this extract were *B. subtilis*, *E. faecalis*, *E. coli* and *P. aeruginosa*. Decoctions of *G. triflora*, *G. macrophylla* and *G. decumbens* tea had slightly lower antimicrobial activity (200–800 μg/mL) with better activity against *E. faecalis* (200 μg/mL). Among the individual compounds gentiopicroside was most active with MIC values of 100–400 μg/mL. The species sensitive to gentiopicroside were *E. faecalis* and *E. coli*. The antibacterial action of loganic acid-6′-*O*-β-d-glucoside was characterised as poorly effective. No inhibition of bacterial growth was shown by the flavones studied, isoorientin and isoorientin-4′-*O*-β-d-glucoside (MIC > 800 μg/mL), as well as water-soluble polysaccharide fractions (MIC > 1600 μg/mL).

**Table 4:** Antimicrobial activity of four bitter gentian tea decoctions, gentiopicroside (G) and loganic acid-6′-*O*-β-d-glucoside (LG), MIC, μg/mL ^a^.

Microorganism	*G. algida*	*G. decumbens*	*G. macrophylla*	*G. triflora*	G	LG	PC ^b^
*B. subtillis*	100	400	400	200	200	400	4.0
*S. aureus*	200	800	400	400	200	>800	4.0
*E. faecalis*	100	200	200	200	100	400	32.0
*E. coli*	100	400	400	200	100	400	2.0
*P. aeruginosa*	100	800	800	400	400	>800	16.0
*C. albicans*	200	>800	>800	800	400	>800	8.0

^a^ Lowest concentration preventing visible grow; ^b^ Positive control—streptomycin, except *C. albicans*—nystatin.

Previous data concerning antimicrobial activity of *Gentiana* herb extractions has indicated close character of activity. Extracts of *G. lutea* leaves and flowers with a high gentiopicroside content (38.85–48.38 mg/g) inhibited growth of 15 microorganisms with MIC values of 120–310 μg/mL [34]. *G. asclepiadea* extract was shown to be active against seven bacteria tested at concentrations ranging from 50–1600 μg/mL [35]. Among the gentian phytochemicals, the compound with the widest spectrum of activity was found to be gentiopicroside [34]. Loganic acid derivatives have also been shown to be effective against pathogenic bacteria and fungi [36]. Concerning information about the antibacterial activity of flavone-*C*/*O*-glycosides, known data differ. No activity of isoorientin against common bacteria and fungi has been reported previously [37]. Lately some activities of flavone-*C*/*O*-glycosides have been demonstrated [38]. Additional information is needed to establish the role of gentian flavonoids in the manifestation of the antibacterial effect of extractions. It deserves special attention because of information about the presence of synergistic interaction of gentian phytochemicals discussed previously [34]. Nonetheless, application of gentian tea decoctions showed an antimicrobial effect against various microorganisms and could be beneficial for treatment of bacterial infections.

#### 2.4.3. Antioxidant Activity

**Figure 3:**
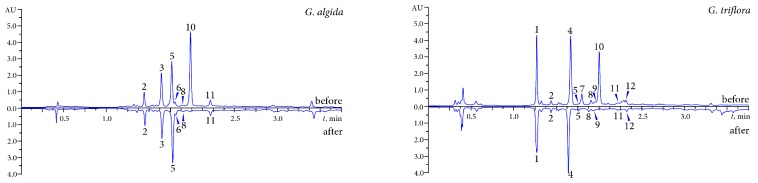
HPLC chromatograms of *G. algida* and *G. triflora* teas decoctions before and after prechromatographic reaction with DPPH-radicals. Compounds: **1**—loganic acid-6′-*O*-β-d-glucoside; **2**—loganic acid; **3**—swertiamarin; **4**—isoorientin-4′-*O*-β-d-glucoside; **5**—gentiopicroside; **6**—sweroside; **7**—mangiferin; **8**—saponarin; **9**—isosaponarin; **10**—isoorientin; **11**—isovitexin; **12**—isoscoparin.

Preliminary characterisation of antioxidant potential was carried out using a DPPH-HPLC procedure, that is HPLC separation of the samples pre-treated with an excess of DPPH^•^ followed by comparison with an HPLC chromatogram of an untreated sample. The reaction between an antioxidant and radical results in the oxidation of the antioxidant, which leads to a decrease of the corresponding peak areas in the chromatograms. The comparison of the HPLC chromatograms of untreated and radical-treated samples allows determination of the most active compounds. Chromatograms of *G. algida* and *G. triflora* tea decoctions spiked with DPPH^•^ radicals are shown in Figure 3, which presents obviously reduced peak areas for some compounds in comparison with untreated samples.

Therefore, only one compound (isoorientin, peak 10) in *G. algida*, *G. decumbens* and *G. macrophylla* and two compounds (mangiferin, peak 7; isoorientin, peak 10) in *G. triflora* demonstrated a visible reduction of the peak area after spiking with DPPH^•^ radicals. Both substances possessed antioxidant activity and they may be concluded as the major active compounds. All detectable iridoids and most flavone-*C*/*O*-glycosides were inactive. The results obtained showed the leading role of isoorientin and mangiferin in free radical scavenging of crude extracts of gentian teas.

The antioxidant properties of the four gentian tea decoctions were evaluated by various tests: 2,2-diphenyl-1-picrylhydrazyl radical (DPPH^•^) scavenging assay; 2,2′-azino-bis(3-ethylbenzthiazoline-6-sulphonic acid) radical (ABTS^•+^) scavenging assay, superoxide radical (O_2_^•−^) scavenging assay; carotene bleaching assay (CBA). All experiments included the determination and comparative estimation of the same antioxidant factors for isoorientin and mangiferin, the main antioxidant compounds of the gentian teas. As can be observed in Table 5, *G. algida* is the most active antioxidant in all assays tested followed by *G. triflora*, *G. macrophylla* and *G. decumbens*. The high antioxidant potential of *G. algida* can be related to it having the highest isoorientin content. Comparative analysis of the two compounds isoorientin and mangiferin confirmed their effectiveness as antioxidants.

**Table 5:** Antioxidant activity of four bitter gentian tea decoctions, isoorientin (^i^O) and mangiferin (Man), IC_50_, μg/mL ^a^.

Method ^b^	*G. algida*	*G. decumbens*	*G. macrophylla*	*G. triflora*	^i^O	Man
DPPH^•^	89.22 ± 3.83 ^ii^	287.91 ± 10.36 ^iii^	278.82 ± 9.87 ^iii^	104.86 ± 3.56 ^ii^	18.67 ± 0.63 ^i^	16.94 ± 0.52 ^i^
ABTS^•+^	101.67 ± 3.15 ^v^	217.03 ± 7.41 ^vi^	187.93 ± 6.76 ^vi^	103.76 ± 3.32 ^v^	14.20 ± 0.42 ^iv^	12.80 ± 0.34 ^iv^
O_2_^•−^	58.63 ± 2.11 ^viii^	> 300	> 300	189.39 ± 6.81 ^viii^	27.69 ± 1.14 ^vii^	21.09 ± 0.97 ^vii^
CBA	32.04 ± 0.89 ^x^	84.15 ± 2.52	55.61 ± 1.56 ^x^	49.37 ± 1.43 ^x^	12.32 ± 0.40 ^ix^	9.62 ± 0.37 ^ix^

^a^ Average of three analyses (±SD); ^b^ DPPH^•^—DPPH^•^ radical scavenging activity; ABTS^•+^—ABTS^•+^ radical scavenging activity; O_2_^•−^—superoxide anion radical scavenging activity; CBA—carotene bleaching assay. All values correspond to mean values ± standard deviation of three replicates. Values with different letters (i–x) indicate statistically significant differences among groups at *p* < 0.05 by one-way ANOVA.

Previously, antioxidant properties of several gentian extractions have been analysed, demonstrating their good ability to scavenge free radicals and inhibit lipid peroxidation. Methanolic extract of the aerial part *G. cruciata* has been shown to be effective in DPPH^•^, ABTS^•+^ and O_2_^•−^ assays with IC_50_ values of 1263.13, 601.15 and 135.73 μg/mL, respectively [39]. The percentage of DPPH^•^ radical scavenging of a MeOH-extract from leaves of *G. asclepiadea*, *G. olivierii*, *S. septemfida* and *G. verna* has been measured as 35.67%–91.70% [40]. Results obtained in the present study indicated a more expressed antioxidant effect of *G. algida*, *G. decumbens*, *G. macrophylla* and *G. triflora* tea decoctions indicating the effectiveness of bitter gentian tea for regulation of the antioxidant status of humans.

## 3. Experimental Section

### 3.1. General Information

#### 3.1.1. Chemicals, Sorbents

Biosupplies Australia Ply Ltd. (Victoria, Australia)—Yariv reagent kit (Cat. No. 100-4); Extrasynthese (Lyon, France)—gentianose (Cat. No. 4319, ≥98%), gentiobiose (Cat. No. 4125, ≥98%), gentiopicroside (Cat. No. 0216, ≥95%*),* isoorientin (Cat. No. 1055 S, ≥99%), isovitexin (Cat. No. 1235 S, ≥99%), loganic acid (Cat. No. 0230 S, ≥99%), saponarin (Cat. No. 1238 S, ≥98%); Sigma-Aldrich (St. Louis, MO, USA)—aspirin (Cat. No. A5376, ≥99%*),* anthrone (Cat. No. 319899, ≥97%), arabinose (Cat. No. A9524*,* ≥98%), 2,2′-azino-bis(3-ethylbenzothiazoline-6-sulfonic acid) diammonium salt (ABTS; Cat. No. Al888, ≥98%), Bradford reagent (Cat. No. B6916), β-carotene (Cat. No. 22040, ≥97%), citric acid (Cat. No. 251275, ≥99%), 3,5-dimethylphenol (Cat. No. 144134, ≥99%), 2,2-diphenyl-1-picrylhydrazyl radical (DPPH^•^, Cat. No. D9132), DMSO (Cat. No. D4540, ≥99.5%), fructose (Cat. No. F2543, ≥98%), galactose (Cat. No. G0750, ≥99%), galacturonic acid (Cat. No. 48280, ≥97%), glucose (Cat. No. G8270, ≥99.5%), glucuronic acid (Cat. No. 851450, ≥99%), hydrogen peroxide (Cat. No. 216763, *ca.* 30%), lithium perchlorate (Cat. No. 431567, ≥99.99%), malic acid (Cat. No. M1210, ≥99%), mangiferin (Cat. No. 06279, ≥98%), mannose (Cat. No. M2069, ≥99%), Mueller Hinton broth (Cat. No. 70192), nystatin (Cat. No. N3503, ≥4400 USP units/mg), oleic acid (Cat. No. 01008, ≥99%), oxalic acid (Cat. No. 75688, ≥99%), perchloric acid (Cat. No. 311421, ≥70%, 99.999% trace metals basis), polyamide for CC (Cat. No. 02395), quinic acid (Cat. No. 138622, ≥98%), resorcinol (Cat. No. 398047, ≥99%), rhamnose (Cat. No. R3875, ≥99%), Sabouraud dextrose agar (Cat. No. S3181), sodium persulphate (Cat. No. 216232, ≥98%), streptomycin sulfate salt (Cat. No. S6501, ≥720 I.U./mg), succinic acid (Cat. No. S3674), sucrose (Cat. No. S7903, ≥99.5%), sweroside (Cat. No. SMB00083, ≥95%), swertiamarin (Cat. No. SMB0008O, ≥95%), tartaric acid (Cat. No. T400, ≥99%), Tween^®^ 80 (Cat. No. P8074), xylose (Cat. No. X1500, ≥99%); Wuhan ChemFaces Biochemical Co., Ltd. (Hubei, China)—isosaponarin (Cat. No. CFN9O 133, ≥95%). Loganic acid-6′-*O*-β-d-glucoside and isoorientin-4′-*O*-β-d-glucoside were isolated previously from *G.* *decumbens* [5].

#### 3.1.2. Equipment

UV-Vis spectrophotometry—SF-2000 UV-Vis-spectrophotometer (OKB Specter, St. Peterburg, Russia); analyt. MC-HPLC—microcolumn chromatograph Econova MiLiChrom A-02 (Novosibirsk, Russia).

### 3.2. Plant Material

The samples of four gentian species were collected in the flowering period in the Buryatia Republic: *G. algida*—Bagdarin (Bauntovskii region, 12.VII.2014, 54°28′26″ N, 113°29′16″ E, voucher specimen No. Gn/h-62/04-11/0714); *G. decumbens*—Mukhorshibir’ (Mukhorshibirskii region, 10.VII.2014, 51°2′14″ N, 107°55′31″ E, voucher specimen No. Gn/h-31/09-24/0714); *G. macrophylla—*Turka (Pribaikal’skii region, 16.VII.2014, 52°55′46″ N, 108°16′31″ E, voucher specimen No. Gn/h-71/06-12/0714); *G. triflora—*Posol’skoye (Kabanskii region, 25.VII.2014, 52°0′20″ N, 106°11′43″ E, voucher specimen No. Gn/h-63/04-09/0714). The species was determined by Prof. T.A. Aseeva (IGEB SB RAS, Ulan-Ude).

### 3.3. Oranoleptic and Nutritional Analysis

#### 3.3.1. Decoction Preparation

The sample of dried milled herb (1 g) was added to distilled water (100 mL), heated on heater plate and boiled 10 min. The mixture was left to stand at room temperature for 15 min, and then filtered under reduced pressure.

#### 3.3.2. Crude Composition

Organoleptic parameters (color, odor, taste) of bitter gentian teas were determined accordingly AHPA guidance on Organoleptic Analysis [41]. Extractives, bitter index and ash were determined accordingly WHO recommendations [42]. The protein content was estimated by Bradford method using BSA as a reference substance [43]. The lipid content was determined by extracting a known weight of dried gentian tea decoction with chloroform–methanol mixture (4:1) using Soxhlet apparatus. Carbohydrate content was determined with spectrophotometric phenol–sulphuric acid method [44]. Energy was calculated according to the following equation: Energy (kcal) = 4 × (g protein + g carbohydrate) + 9 × (g lipids). Free amino acids content was determined with ninhydrin method [42].

#### 3.3.3. Free Sugars Composition

Free sugars were determined by a Milichrom A-02 microcolumn HPLC system, using Separon 5-NH_2_ column (1 × 60 mm, Ø 1 μm; Tessek Ltd.; Prague, Czechia), column temperature was 20 °C. Mobile phase was acetonitrile–water 75:25. The injection volume was 1 μL, and elution was at 100 μL/min. Detector wavelength was 190 nm. Reference sugars retention times (t, min): fructose (11.73), glucose (13.24), sucrose (17.45), gentiobiose (18.46), gentianose (19.94). The results were expressed in mg per 100 mL of decoction. HPLC chromatograms of the reference carbohydrate mixture and gentian tea samples are presented as the Supplementary Material (Supplementary Material Figure S1).

#### 3.3.4. Organic Acids Composition

Organic acids were determined by a Milichrom A-02 microcolumn HPLC system, using a ProntoSIL-120-5-C18 AQ column (1 × 70 mm, Ø 5 μm; Metrohm AG; Herisau, Switzerland), column temperature was 35 °C. Eluent A was 0.2 М LiClO_4_ in 0.01 M HClO_4_ and eluent B was acetonitrile. The injection volume was 1 μL, and elution was at 50 μL/min. Gradient programme: 0–20 min, 1% B; 20–25 min, 1%–10% B. Detector wavelength was 210 nm. Reference organic acids retention times (t, min): oxalic (2.17), tartaric (4.03), malic (5.93), citric (9.74), succinic (11.02), quinic (12.22). The results were expressed in mg per 100 mL of decoction.

### 3.4. MC-RP-HPLC-UV Quantification of Phytochemicals in Bitter Gentian Teas

MC-RP-HPLC-UV experiments were performed on an Econova MiLiChrom A-02 microcolumn chromatograph (Novosibirsk, Russia) coupled with a UV-detector, using a ProntoSIL-120-5-C18 AQ column (1 × 50 mm, Ø 1 μm; Metrohm AG; Herisau, Switzerland); the column temperature was 35 °C. Eluent A was 0.2 М LiClO_4_ in 0.006 M HClO_4_ and eluent B was acetonitrile. The injection volume was 1 μL, and elution was at 600 μL/min. Gradient program: 0–2.5 min, 5%–35% B; 2.5–4 min, 35%–70% B. UV-detector wavelengths were 254 nm (iridoids) and 334 nm (flavonoids, mangiferin). Decoctions of gentian herb teas prepared accordingly a protocol described in Section 3.3.1 were filtered through a 0.22 μm PTFE syringe filter before injection into the HPLC system for analysis. Stock solutions of standards were made by accurately weighing 1 mg of loganic acid, loganic acid-6′-*O*-β-d-glucoside, swertiamarin, sweroside, gentiopicroside, isoorientin, isoorientin-4′-*O*-glucoside, isovitexin, saponarin, isosaponarin, isoscoparin, and mangiferin and dissolving it in 20 mL of methanol/DMSO in a volumetric flask. The appropriate amounts of stock solutions were diluted with methanol in order to obtain standard solutions containing 0.25–1.00 mg/mL. As all the compounds used for quantification were well-separated in experiment conditions mixtures of standards were analyzed. Prepared solutions were stored at 4 °C for no more than 72 h. The results are presented as mean values ± SD (standard deviations) of the three replicates.

### 3.5. Polysaccharide Analysis

The sample of dried milled herb (100 g) was added to distilled water (10 L), heated on a boiled water bath (1 h) and after cooling to room temperature water extract was filtered under reduced pressure and concentrated down *in vacuo* to 200 mL. The concentrated residue was mixed with 95% ethanol (1:5) and after 2 h the precipitate was centrifuged at 3000 *g*. The crude polysaccharide fraction was redissolved in 200 mL of water. The Sevag method [45] was used for deproteinisation, and was followed by dialysis for 48 h against distilled water using dialysis tubes with an MW-cut off of 2 kDa (Sigma-Aldrich, St. Louis, MO, USA). The non-dialysed part was loaded on to a KU-2-8 cation-exchange resin column (H^+^-form, 200 g; Closed Joint-Stock Company Tokem, Kemerovo, Russia) which was eluted with 2 L of distilled water. Eluate was concentrated *in vacuo* up to 200 mL and then liophylized. WSPF obtained were off-white powders.

Total carbohydrate content (TCC) was determined with the spectrophotometric phenol-sulphuric acid method [44]; content of uronic acids (UA) was estimated by the 3,5-dimethylphenol method calculated as galacturonic acid [46]; and the proteins were determined by the Bradford method using Coomassie G250 [43]. The optical rotation was measured at 40 °С for 1% sample solutions in 0.5% KOH using a SM-3 polarimeter (Zagorskii Optiko-Mekhanicheskii Zavod, Zagorsk, Russia) equipped with a 1 dm cell. Reactions of WSPF solutions with iodine, resorcinol and Yariv reagent were performed accordingly [47,48,49]. IR spectra were registered in a spectral range of 4000–600 cm^−1^ using a FT-801 infrared Fourier spectrometer (Simex, Novosibirsk, Russia) coupled with single reflection ATR device. The hydrolysis procedure and HPLC analysis conditions of the released products were as described by us previously [50].

### 3.6. Biological Activity Assays

The experimental procedures relating to the animals were authorised by the Institute of General and Experimental Biology’s Ethical Committee (protocol No. LM-0324, 27.01.2012) before starting the study and were conducted under the internationally accepted principles for laboratory animal use and care.

#### 3.6.1. Carrageenan-Induced Paw Edema in Male Rats

Sprague Dawley rats weighing *ca*. 200–250 g were purchased from the “Pushchino” Laboratory Animal Breeding House (Moscow, Russia), and were kept in polyethylene boxes under a controlled temperature of 25 °C, humidity of 55% and a 12 h light/dark cycle, with free access to standard food and water. Animals were weighed and randomised into 14 groups of 12 animals each. Control group: water treatment; positive drug group: aspirin treatment (200 mg/kg); four bitter gentian tea groups: intragastrically administered three doses (100, 200, 400 mg/kg), once a day for seven days. For the determination of effects on acute inflammation, the carrageenan-induced paw oedema model described by Yesilada *et al.* [51] was employed with modifications. One hour after the last delivery, 100 μL of a 1% solution of carrageenan in 0.9% physiological saline was injected subcutaneously into the subplantar region of the right hind paw. The paw volume was measured with a water displacement plethysmometer 37140 (Ugo Basile, Varese, Italy) at 1–4 h after induction of inflammation. The percentage inhibition of paw volume in the drug-treated group was compared with the control group. Inhibitory activity was calculated according to the following formula described in the literature [52]:

(1) $$I\left(\%\right)\;=\;\left[\left(V_{S}-V_{0}\right)/V_{0}\right]\;\times \;100\%$$

where *I* is the inhibition of oedema, *V*_S_ is the displacement volume after carrageenan administration and *V*_0_ is the displacement volume before carrageenan administration.

#### 3.6.2. Antimicrobial Activity

The bitter gentian tea decoctions and individual compounds were individually tested against six microorganisms. The following microbial strains were used in this research: *Bacillus* *subtilis* (ATCC 6633), *Staphylococcus aureus* (ATCC 6538P), *Enterococcus faecalis* (ATCC 12952), *Escherichia coli* (ATCC 25922), *Pseudomonas aeruginosa* (ATCC 27835); the test fungus was *Candida albicans* (ATCC 10231). All test microbial strains were obtained from the Institute of Biochemistry and Physiology of Microogranisms (Pushchino, Russia). Bacterial strains were cultured overnight at 37 °C in nutrient agar and fungi were cultured on Sabouraud dextrose agar at 28 °C for 5 days. The material obtained was suspended in sterile water with a concentration of 5 × 10^5^ CFU/mL for bacterial strains and 3 × 10^4^ CFU/mL for *C. albicans*. The MICs of the bitter gentian tea decoctions and individual compounds against tested microorganisms were determined based on a microdilution method in 96 multi-well microtiter plates [53] with slight modification as described by Mihailović *et al.* [35]. All tests with bacterial strains were performed in Mueller-Hinton broth (MHB), and for *C. albicans* Sabouraud dextrose broth (SDB) was used. A volume of 50 µL stock solutions of bitter gentian tea decoctions or individual compounds (in 50% DMSO, 1.6 mg/mL) were added into the first row of the plate. To all other wells 50 µL of MHB or SDB was added. A volume of 50 µL from the first test well was pipetted into the second well of each microtiter line, and then 50 µL of scalar dilution was transferred from the second to the ninth well. To each well, 10 µL of resazurin indicator solution (6.5 mg/mL in sterile distilled water) and 30 µL of nutrient broth were added. Finally, 10 µL of bacterial suspension (5 × 10^5^ CFU/mL) and *C. albicans* spore suspension (3 × 10^4^ CFU /mL) was added to each well. In tests with fungi instead of resazurin indicator solution, 10 µL of SDB was added. For each strain, the growth conditions and the sterility of the medium were checked. Standard antibiotic streptomycin was used to control the sensitivity of the tested bacteria, whereas nystatin was used as control against *C. albicans*. Plates were placed in an incubator at 37 °C for 24 h for the bacteria and at 28 °C for 48 h for *C. albicans*. The visual growth was then assessed. Any colour change from purple to pink or colourless was recorded as positive. The lowest concentration at which there was no observed colour change was taken as the MIC value for bacterial strains, and the lowest concentrations without visible growth were defined as concentrations that completely inhibited fungal growth. All tests were repeated in triplicate.

#### 3.6.3. Antioxidant Activity

The DPPH-HPLC-UV procedure was realized as described previously [54]. The DPPH^•^ radical scavenging activity (DPPH^•^) was assessed as described by Asker and Shawky [55]; the ABTS^•+^ radical scavenging activity (ABTS^•+^-SA) was measured using the method of Ding *et al.* [56]; the determination of superoxide anion scavenging activity (O_2_^•−^-SA) was measured in phenazine methosulphatenicotinamide adenine dinucleotide-nitroblue tetrazolium systems using the method of Ozen *et al.* [57]; β-carotene bleaching assay (CBA) was performed in β-carotene-oleic acid-DMSO-H_2_O_2_-system [58].

### 3.7. Statistical Analysis

Statistical analyses were performed using a one-way analysis of variance (ANOVA), and the significance of the mean difference was determined by Duncan’s multiple range test. Differences at *p* < 0.05 were considered statistically significant. The results are presented as mean values ± SD (standard deviations) of the three replicates.

## 4. Conclusions

In conclusion, the present study demonstrated that four gentian teas (*G. algida*, *G. decumbens*, *G. macrophylla*, *G. triflora*) used in Siberia could be distinguished plant products in regard to their nutritional and chemical profiles, as well as their potential medical uses. The results showed the equilibrated composition of the nutraceutical compounds and specific phytochemicals in the gentian herb decoctions. The gentian teas preparations demonstrated expressed anti-inflammatory, antimicrobial and antioxidant activities the most significant for *G. algida* tea. Chemical features as a high gentiopicroside and isoorientin content may be responsible for the pharmacological effectiveness. The gentian teas bioactivity can be used in the food industries and medicine.

## Supplementary Materials

Supplementary materials can be accessed at: http://www.mdpi.com/1420-3049/20/11/19674/s1.
