# Supplementary material for: Bitter Gentian Teas: Nutritional and Phytochemical Profiles, Polysaccharide Characterisation and Bioactivity
Source: Molecules. 2015 Nov 5;20(11):20014–30. doi: 10.3390/molecules201119674 (PMC6331966; doi:10.3390/molecules201119674)
Supplement: Supplementary file 1 [file molecules-20-19674-s001.pdf]

molecules

MDPI

Supplementary Material

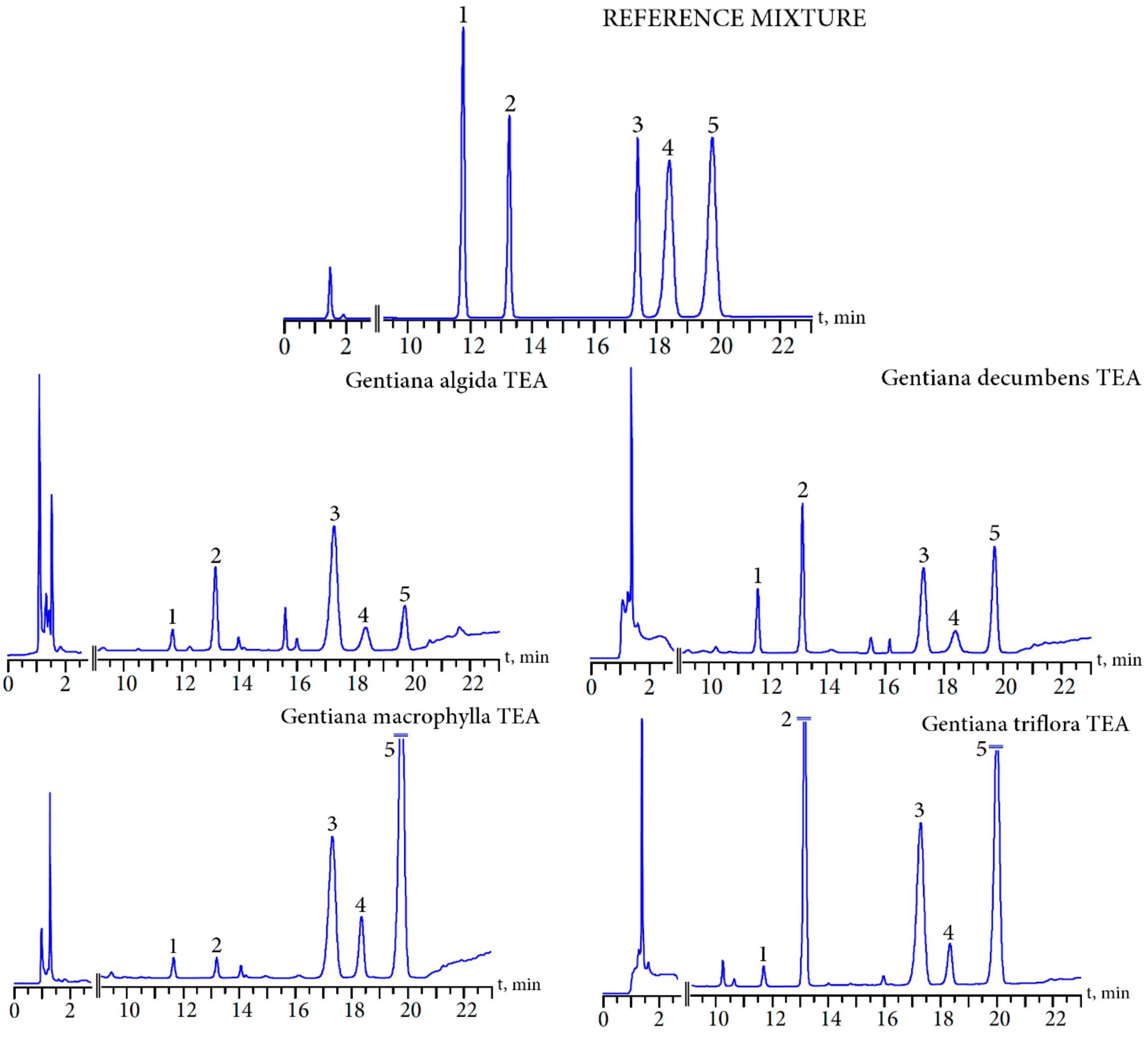

Figure S1. HPLC chromatograms of the reference mixture of carbohydrates and *Gentiana algida* tea decoction. Compounds: **1**—fructose; **2**—glucose; **3**—sucrose; **4**—gentiobiose; **5**—gentianose.

HPLC conditions. Apparatus—HPLC Milichrom A-02 microcolumn system; column—Separon 5-NH_2 (1 × 60 mm, Φ 1 μm; Tessek Ltd.; Prague, Czechia); column temperature—20 °C; mobile phase—acetonitrile–water 75:25; injection volume—1 μL; elution rate—100 μL/min; detector wavelength—190 nm. Concentrations of the compounds in the reference mixture: fructose—20 μg/mL; glucose—15 μg/mL; sucrose—20 μg/mL; gentiobiose—25 μg/mL; gentianose—25 μg/mL.
